# Reply to Letter to the Editor for “Two-year Results of an Emergency Department Night Shift Buyout Program”

**DOI:** 10.5811/westjem.69561

**Published:** 2026-11-02

**Authors:** Charlotte W. Croteau, Joshua N. Goldstein, Lauren Nentwich, Ali S. Raja, Michael VanRooyen, Joshua J. Baugh

**Affiliations:** Harvard Medical School, Massachusetts General Hospital, Department of Emergency Medicine, Boston, Massachusetts

Dear Editor:

We thank the authors for their thoughtful letter and for engaging so seriously with the ethical dimensions of our night shift buyout program. The distinction they draw between egalitarian and equitable systems is a useful one, and we agree that in an idealized group of emergency medicine faculty, nights, holidays, and weekends would simply be shared evenly across all members.

In practice, however, this ideal rarely survives contact with a real department. Faculty carry medical conditions, family obligations, and life circumstances that departments routinely and reasonably accommodate. In some groups, exemption from night shifts is treated as a perk of seniority; given the well-documented disproportionate physiologic impact of night work on older clinicians, many departments simply let senior faculty opt out entirely, which has the effect of concentrating more nights on the most junior members. We suspect that most emergency departments already operate some form of unequal or inequitable night shift distribution, whether it is named as such or not. Nursing, a no less demanding profession, has long compensated nights differently than days and lives with the same equity tensions the authors raise here.

Viewed against that backdrop, we believe our program offers not only equality of opportunity but, in practice, more equity than many systems that may aspire to an unattainable ideal and end up instead with a patchwork of informal accommodations that are typically less equitable—and certainly less transparent—than a system with an explicit and uniform price. We would also candidly note that our current program is considerably more equitable than the one it replaced, in which night shift assignment tracked directly with age and academic rank, both of which historically skewed heavily male in our department.

We agree with the authors that organ donation is a higher-stakes case for the ethics of commodification than night shift scheduling, and we are not certain that perfect equity should be the primary goal of a night shift system in the first place. As the authors themselves observe, increased autonomy and control over one’s schedule may be the greatest advantage of a buy-out model, acknowledging that scheduling needs and preferences are far from uniform across different emergency physicians, or even within the same physician at different stages of life. We therefore placed a high priority on autonomy and choice by design, not as an incidental benefit.

This perhaps raises a pertinent question for the authors in return: What alternative would they propose? One common model exclusively exempts older or more senior faculty from nights, which raises many equity concerns, particularly if older and more senior faculty differ in characteristics from the rest of the group. Given recently documented trends of women emergency physicians leaving the field earlier than men,^1^ an age-based model in particular risks asking women to work nights for a longer portion of their careers than men. Another option is a fixed, non-negotiable allotment of nights distributed equally to all faculty, with no mechanism to reduce that number. Under that model, no faculty member is ever worse off in night burden than any peer—this appears the most equitable version on the surface. However, this model would ask older emergency physicians to continue working nights until retirement, which prior research suggests is likely not beneficial for patient care quality or career longevity. It would also result in the loss of autonomy and flexibility that the authors credit our program with achieving. Such a model also presumes it is equitable to make the same requirements of all emergency physicians regardless of life circumstances; it is easy to envision this not necessarily being true.

We share the authors’ view that ethical and optimal distribution of undesirable shifts is fertile ground for further analysis. Productive future efforts might include a multicenter analysis of scheduling approaches across various settings, along with measured outcomes, to inform the program choices that emergency departments across the country will make in years to come.
